# Patient and caregiver perspectives on guideline adherence: the case of endocrine and bone health recommendations for Duchenne muscular dystrophy

**DOI:** 10.1186/s13023-019-1173-7

**Published:** 2019-08-20

**Authors:** Brian Denger, Kathi Kinnett, Ann Martin, Sean Grant, Courtney Armstrong, Dmitry Khodyakov

**Affiliations:** 1 Parent Project Muscular Dystrophy, 401 Hackensack Avenue, 9th Floor, Hackensack, NJ 07601 USA; 2 RAND Corporation, 1776 Main Street, PO Box 2138, Santa Monica, CA 90407-2138 USA; 3 RAND Corporation, 1885 Mission Street, Suite 300, San Francisco, CA 94103 USA; 4 Department of Social & Behavioral Sciences, Indiana University Richard M. Fairbanks School of Public Health, 1050 Wishard Blvd, RG 6046, Indianapolis, 46202 IN USA

**Keywords:** Duchenne muscular dystrophy, ExpertLens, Guideline adherence, Modified-Delphi, Patient engagement

## Abstract

**Background:**

Clinical care guidelines are typically developed by clinicians and researchers. Including patient and caregiver voices in guideline development may help create guidelines that are more useful for patients and consequently improve their guideline adherence. Although there is substantial research on the factors that affect providers’ adherence to guidelines, there is less research on the factors that affect patients’ compliance with guideline recommendations, especially among those with rare disorders.

The purpose of this study is to explore factors that are likely to affect patient/caregiver adherence to endocrine and bone health recommendations for Duchenne Muscular Dystrophy (DMD). To do so, we used qualitative data collected as part of the study designed to develop, implement, and evaluate a new online, modified-Delphi approach to engaging patients with rare diseases and their caregivers in guideline development, using care guidelines for DMD as a case study.

**Methods:**

We thematically analyzed qualitative data collected from 95 adults with DMD and their caregivers who participated in at least one round of our online modified-Delphi panel process. Participants rated and commented on the patient-centeredness of 19 recommendations about vertical growth, weight management, bone health, and delayed puberty included in the 2018 DMD care considerations. Patient-centeredness was operationalized as the importance and acceptability of care recommendations.

**Results:**

Thematic analyses revealed six factors that affect guideline adherence from the patient/caregiver perspective: content and format of recommendations, patient and provider characteristics, and social and financial factors.

**Conclusions:**

This study used a novel approach to exploring patient and caregiver perspectives on factors that may affect guideline adherence. The six factors identified by DMD patients and caregivers are similar to the factors affecting provider adherence and are not limited to DMD. Understanding consistency between provider- and patient/caregiver-identified barriers to following guideline recommendations can lead to developing more successful interventions for increasing guideline adherence.

**Electronic supplementary material:**

The online version of this article (10.1186/s13023-019-1173-7) contains supplementary material, which is available to authorized users.

## Background

Clinical care guidelines have traditionally been developed by clinicians and researchers recognized as experts for the targeted condition [1]. Guideline development groups use available medical evidence and their professional expertise to rate the appropriateness and necessity of different treatment options for a typical patient with a specific diagnosis [2]. But treating patients and families merely as consumers of these materials fails to recognize the needs and preferences of individuals with expertise in the “lived experience” of a given medical condition. These individuals know firsthand which practices are likely to be deemed trustworthy and followed by patients and caregivers, and which practices are likely to be judged unacceptable [3]. Patient engagement may help develop guidelines that are trustworthy, useful for patients across the spectrum of the diagnosis, and likely to be adhered to [4].

Including patient and caregiver voices when developing clinical care guidelines is part of the growing trend to support a patient and family-centered practice [5, 6]. Advocates include the Institute of Medicine [7], Guidelines International Network [8], and the National Institute for Clinical Excellence [9], among others. Patient and caregiver perspectives can be discerned by examining the literature or by directly soliciting their input. However, the literature may not report patient-reported or patient-preferred outcomes for a given condition [10], especially if it is a rare disorder. Therefore, directly engaging a broad cross section of patients and their caregivers has special value, particularly in the case of rare diseases.

Nonetheless, it is often difficult to recruit patients, especially with rare diseases [10], and to engage large numbers of patients in this process [3]. The ability to assemble large groups in rare disease communities may be hindered by participant availability, high cost, and scheduling challenges; ensuring that different perspectives are well-represented adds another barrier. One way to overcome these challenges is to use web-based tools [11] that help individuals rate and comment on guideline recommendations as a way to determine their perceived patient-centeredness [12].

The Patient-Centered Outcome Research Institute (PCORI) funded our project to develop, implement, and evaluate an online approach to engaging patients with rare diseases and their caregivers in guideline development. Our project team consisted of researchers from RAND, clinicians and genetic councilors from Parent Project Muscular Dystrophy (PPMD), and individuals with Duchenne Muscular Dystrophy (DMD) and their caregivers. Our approach allows patients and caregivers to engage in guideline development in a way that is convenient, rigorous, and consistent with how clinicians participate in guideline development. Instead of commenting on clinical appropriateness and necessity of different care options, patients and caregivers provide direct input on the patient-centeredness of care guidelines by rating and commenting on their importance and acceptability for a typical patient/caregiver living with Duchenne using an online modified-Delphi approach [12].

In this paper, we use thematic analyses of qualitative data collected as part of our larger project that developed a new approach to patient and caregiver engagement in guideline development to describe factors that affect the perceived patient-centeredness of the 2018 DMD care considerations for endocrine and bone health care [13–15]. We argue that the factors affecting patient and caregiver perceptions of guideline importance and acceptability—the two key factors that make up patient-centeredness of care guidelines—are likely to influence whether patients and caregivers comply with guideline recommendations.

Our study contributes to the body of literature on guideline adherence, which typically focuses on factors that affect providers’ use of guidelines [16, 17]. Learning about concerns that families living with Duchenne may have could help providers better educate their patients about pros and cons of different treatment options and initiate a shared decision-making process about treatments. By working together, providers and Duchenne families can help increase guideline adherence, which has been low not only in the US [16], but also in other countries [18].

## Methods

For our larger project, we used an embedded mixed-methods study design to determine perceived patient-centeredness of 2018 DMD care considerations [19] using an online modified-Delphi approach [12]. Quantitative analysis of rating data helped us determine participants’ ratings of patient-centeredness; thematic analyses of their comments helped explain the factors that may affect their ratings. Results of our quantitative analyses of patient-centeredness ratings will be reported separately. This paper uses qualitative analysis to identify factors that may affect guideline adherence from the patient and caregiver perspective.

### Participants

For the larger study, we recruited 27 adults with Duchenne and 95 caregivers from the PPMD Duchenne Registry, the largest curated patient and caregiver registry in the U.S. for Duchenne and Becker Muscular Dystrophy [20]. We randomly assigned participants to one of two panels comprising both patients and caregivers. We balanced panel composition by using stratified randomization on caregivers’ educational attainment, ambulatory status of the individual with Duchenne, and the distance to the closest PPMD Certified Duchenne Care Center [21]; community experience and/or literature suggested distance might affect guideline adherence [16, 17]. Both panels were conducted at the same time and followed identical protocols [12].

### Design

We used a novel online modified-Delphi platform called ExpertLens [22] and the RAND/UCLA Appropriateness Method (RAM) [2] (the method that clinicians used to develop the DMD care considerations) to determine how individuals with Duchenne and their caregivers viewed the patient-centeredness, operationalized as importance and acceptability (see below), of 19 recommendations for endocrine care (vertical growth, weight management, and pubertal development) and bone health. These sections of the 2018 DMD care considerations were developed without patient input. Moreover, previous research identified wide variation in providers’ adherence to earlier versions of many of these recommendations [16, 17].

A three-round data collection took place between March 12 and April 24, 2018, a month after the 2018 DMD care considerations were published. In Round 1, participants used 9-point Likert scales to rate the importance and acceptability of each recommendation for a typical family living with Duchenne. We defined importance as the extent to which “a clinical reason for recommendation is likely to be consistent with the preferences, needs, and values of Duchenne families in general.” We defined acceptability as the extent to which “the process of following a given recommendation is likely to be consistent with available resources (e.g., time and finances) and with the ethical standards of Duchenne families in general.” Participants were encouraged to explain their ratings using open text boxes. In Round 2, participants reviewed their own and their group’s ratings and engaged in an online discussion. Study investigators and one parent caregiver of two sons living with Duchenne, moderated discussions to promote active engagement, tease out participant opinions, ask clarification questions, and direct participants to additional resources to facilitate their understanding of care recommendations. Every effort was made to avoid inflecting bias by using benign prompts, such as asking what the group thought about a particular point or comment. In Round 3, participants were encouraged to revise their Round 1 ratings in light of Round 2 discussion. Participants received a $50 gift card for completing each round.

### Data analysis

Our data analysis consisted of three stages. We first looked at participation rates across the Delphi rounds. Because attrition is a common problem in Delphi studies [23], we used Fisher’s exact test to see if there are statistically significant differences between participants and non-participants in different rounds. This test helps identify any response bias by looking at associations between participation status and demographic characteristics to determine if non-participants differ from participants.

Next, we analyzed the rating data to determine recommendations’ patient-centeredness, which will be reported separately. To do so, we used RAM to determine consensus on the importance and acceptability of each recommendation [2]. We applied this methodology to Round 3 ratings (see our research protocol [12] for a step-by-step description of how consensus was determined). We considered a recommendation to be patient-centered only if it was deemed important and acceptable by both panels. Additional file 1 list patient-centeredness status of all 19 recommendations.

Finally, to address the aim of this paper, we thematically analyzed Round 1 explanations of ratings and Round 2 discussion commets to illustrate the factors that affected participants’ perceptions of recommendations’ importance and acceptability and to identify the factors that are likely to affect guideline adherence from the patient/caregiver perspective. A team of coders trained by the principal investigator (PI) independently coded all qualitative comments inductively to identify emergent themes that could be used to explain why a certain care consideration was or was not considered important or acceptable. All coding results were reviewed by the PI to ensure consistency in how the codebook was applied, as well as by a clinician, a genetic counselor, and a caregiver to ensure the comments were correctly interpreted. Rare disagreements among the reviewers were discussed until resolved, as recommended in the literature [24]. Finally, a caregiver representative and a clinician reviewed each theme to determine whether it could affect guideline adherence. Once the coding was complete, the team compared the list of factors that patients and caregivers think may affect guideline adherence with the factors that the literature suggests affect provider compliance with clinical guidelines. We focus on these comparisons in the discussion section of this manuscript.

## Results

Out of 122 invited participants, 95 (78%) participated in at least one round by either answering at least one rating question or reviewing/making comments during discussion. Of the 95 participants, 88 (93%) participated in Round 1; 74 (78%) participated in  Round 2 and, of these, 55 (74%) posted a total of 1201 comments (M = 21.8, SD = 34.2, Range: 1–209); and 56 (59%) participated in Round 3.

The majority of those who participated in at least one round were parents or caregivers (75%); most were female (62%) and white (91%). About two-fifths  of study participants reported living within a 50-mile radius from a clinic where individuals with DMD receive neuromuscular care. There were no statistically significant differences in demographic characteristics of those who participated and those who did not participate in different rounds (see Table 1). There were no statistically significant differences between demographic characteristics of participants and non-participants in different rounds as measured by Fisher’s exact test.

**Table 1:** Participant Characteristics

	Participated in at least 1 round % (*n* = 95)	Participated in Round 1 (*n* = 88)	Participated in Round 2 (either viewed or posted) (*n* = 74)	Participated in Round 3 (*n* = 56)
Participant Type				
Caregiver	71 (75%)	65 (74%)	58 (78%)	43 (77%)
Individual with DMD	24 (25%)	23 (26%)	16 (22%)	13 (23%)
Gender				
Female	59 (62%)	53 (60%)	47 (64%)	35 (63%)
Male	36 (38%)	35 (40%)	27 (36%)	21 (37%)
Hispanic/Latino/Spanish^a^				
Yes	4 (4%)	2 (2%)	3 (4%)	1 (2%)
No	90 (96%)	85 (98%)	70 (96%)	54 (98%)
Race				
White	86 (91%)	82 (93%)	67 (91%)	52 (93%)
Black/African American	1 (1%)	1 (1%)	1 (1%)	0 (0%)
Asian	4 (4%)	3 (3%)	4 (5%)	4 (7%)
Multi-race	1 (1%)	0 (0%)	1 (1%)	0 (0%)
Other	3 (3%)	2 (2%)	1 (1%)	0 (0%)
How far do you usually travel to receive neuromuscular care?^a^				
< 50 miles	38 (41%)	37 (44%)	30 (41%)	25 (46%)
50–99 miles	22 (24%)	20 (24%)	16 (22%)	12 (22%)
100–249 miles	17 (19%)	13 (15%)	14 (19%)	8 (15%)
≥ 250 miles	15 (16%)	15 (18%)	13 (18%)	9 (17%)

There were no statistically significant differences between participants and non-participants in different rounds as measured by Fisher’s exact test

^a^ Not all participants provided responses to this question

Of the 19 care considerations included in this study, 12 met criteria of patient-centeredness (i.e., both panels considered them to be important and acceptable). See Additional file 1 for a list of recommendations. Of these, four pertained to vertical growth, three to weight management, three to bone health, and two to puberty. Panels disagreed on the importance and/or acceptability of four recommendations and considered the remaining three to be of uncertain importance and acceptability. A more detailed description of these findings will be published separately.

### Patient-centeredness of and factors affecting adherence to bone health recommendations

Although all recommendations for bone health and weight management were deemed patient-centered, our participants identified several issues that may affect adherence, especially for certain types of patients. To illustrate, some participants argued that bone health assessments are “more important for ambulatory patients because they’re more susceptible to falling … [and because] an ambulatory patient is less likely to have fragile bones.” Nonetheless, one caregiver noted that his/her “ambulatory son has rather fragile bones - I’m guessing mostly due to steroids.” An individual with DMD added that “bone density goes down over time because we don’t walk,” which increases the risk of fractures. Ultimately, participants agreed that bone health recommendations are “pretty important even for non-ambulatory [patients] if they are at such a risk for compression fractures.” However, this discussion suggests that providers should educate patients and caregivers on the importance and benefits of early detection of bone health issues.

While participants considered X-rays and DEXA scans used to assess bone health as minimally invasive, safe, and painless, some raised concerns about acceptability of these procedures, citing difficulties in transferring boys and young men to an x-ray table, the ability of those who have disease-related joint deformities to lie flat on a table during procedures, the ability of DEXA scans to produce accurate results for pediatric patients, and the costs of DEXA scans that may not always be covered by insurance. As one caregiver noted, “similar to the spine X-ray the DEXA scan is difficult for patients who are non-ambulatory, heavy, and have contractures of the joints. Those administering the test need to have a great deal of patience and problem-solving skills.” Another stated that although “quick, safe, and painless...the insurance coverage and cost [of these procedures is of concern].”

Moreover, there was extensive discussion of pros and cons of IV bisphosphonate treatments for individuals who had fractures or bone loss. A caregiver commented: “My boys get Pamidronate infusions every 4 months, and they don’t really have any side effects.” An adult with DMD stated: “I understand this [recommendation] is to treat osteoporosis and to prevent further bone loss. I believe fighting bone loss is very important, and you want to do all you can. Especially for later in life when osteoporosis can make transferring the individual difficult.” Some participants, however, raised concerns about off-label use of IV bisphosphonates for treating vertebral fractures and bone loss in children: “The restriction for children in many countries would make me want to learn more before pursuing this course of treatment.” High drug costs and potential side effects were of concern as well. Finally, both individuals with DMD and their caregivers stated that because this treatment requires injections, it may be acceptable only if a child is not afraid of needles: “Some families/patients may be fearful of IV. Depending on the frequency, it may be a difficult experience or feel inconvenient.”

To summarize, discussions about bone health recommendations illustrate that patients and caregivers may agree that a recommendation is very important, but may have concerns about the process of following it. In such a situation, providers may need to address patient/caregiver concerns, such as potential discomfort, early in treatment and search for ways to mitigate them. Doing so can help increase guideline adherence.

### Patient-centeredness of and factors affecting adherence to weight management recommendations

Participants endorsed all weight management recommendations by confirming the importance and acceptability of both diet and exercise for individuals with DMD, especially during glucocorticoid use. As a caregiver stated: “[Diet is] very important because steroid use can make your weight increase, so keeping a healthy diet will help lower the weight.” Participants discussed issues related to being overweight and underweight, both of which can negatively affect quality of life. On the one hand, boys with DMD can gain weight because of their inability to exercise, which may make it difficult to care for them and may cause further social ostracism. On the other hand, they may develop problems with swallowing solid food as they get older, which can lead to weight loss. Participants suggested taking protein shakes regularly to help boys increase body mass. They also discussed how spinal fusion surgery can negatively affect boys’ abilities to feed themselves, exercise, and attend school full time due to the difficulties of staying in a wheelchair for extended periods of time and urinating into a bottle. The discussion of weight management recommendations suggests that providers should highlight the link between weight gain and quality of life, while also trying to help Duchenne families find practical solutions to logistical challenges they face daily.

In discussing acceptability of physical activity recommendations, participants raised concerns about insurance coverage for physical therapy and noted the need for a more specific guidance about the type of exercise that should be done or avoided, when exercise should be stopped, and how play activities favored by boys could be adapted to their abilities as their functions deteriorate. Participants stressed that “it’s good to encourage it, educate people on proper stretches, exercises that are not damaging to muscles or if possible to help with strength or flexibility for those with Duchenne, and how often to do it so that healthy weight is maintained.” An individual with DMD suggested that “overexertion can speed up the muscle weakness, so it’s important that it’s done under the supervision of a physical therapist with expert knowledge of Muscular Dystrophies.” Indeed, finding the right type of exercise while not overdoing it is key. Participants noted that stretching and swimming might be the most appropriate physical activities for boys with DMD and emphasized the importance of enjoying physical activities, while reducing the level of unnecessary fatigue (e.g., getting to a playground in a wheelchair and then getting out to play). Caregivers noted that it is important to involve the whole family to ensure that “no one is singled out.”

In summary, participants felt that the physical activity recommendations were patient-centered; however, they viewed them as too vague and wanted examples of physical activities that may work well for boys with Duchenne. This information can help providers suggest a range of activities that Duchenne families can explore to facilitate their adherence to these recommendations.

### Patient-centeredness of and factors affecting adherence to vertical growth recommendations

Only four vertical growth recommendations, all of which focused on assessing and identifying growth delays, met our patient-centeredness criteria. Some caregivers noted that assessing growth is important, but treating growth delays may not be: “Height is important to most of the guys (and therefore their families), but it is not likely that their height will be comparable to healthy boys; and actually, having a smaller stature may benefit them and their mobility.” Indeed, many participants who gave growth assessment recommendations a low rating commented that height is not as important as other issues: “Vertical growth delays probably isn’t as major of an issue as say delayed puberty or osteoporosis.”

The only assessment-related recommendation that did not meet our patient-centeredness criteria was identifying growth impairment in boys between 13 and 18 years of age. Some participants noted that doing so at this age may be already too late. Our analysis suggests that a child’s age may be an important factor that can affect guideline adherence, so finding the right time to discuss vertical growth delays may be key to adherence.

Participants rated recommendations to use recombinant human growth hormone (rhGH) therapy to treat growth delays as not patient-centered due to uncertain ratings of importance and acceptability. Panelists debated whether being short really required rhGH treatments. Both caregivers and individuals with DMD raised concerns about potential side effect and uncertain efficacy. As an individual with DMD put it, rhGH comes with “high risk, [and] little to no reward.” Another commented on “the inconclusive evidence of effectiveness.” Caregivers responded similarly: “Now that I think about it more and read others’ comments, worry about side effects it could have and pain it can cause. My son wanted to be taller, but when he found out that it would be injections, he didn’t want it. It also wasn’t recommended because he had normal growth hormone. We are okay if he’s shorter. There are more important issues.” Participants’ views on the use of growth hormone therapy appeared to mirror the opinion of the clinical experts who developed these care considerations. The recommendation *is not to routinely use rhGH in patients without documented growth hormone deficiency* due to inconclusive evidence of its effectiveness.

In summary, the rhGH discussion illustrates the importance of being transparent about pros and cons of a particular treatment and shows that vertical delays are a preference-sensitive topic. While some patients may find it important to address growth delays and acceptable to use rhGH to treat them, others may prefer not to treat them at all or use other means. Therefore, it is important for providers to engage individuals with DMD and their caregivers in a shared decision-making on this topic as a strategy for increasing guideline adherence.

### Patient-centeredness of and factors affecting adherence to puberty recommendations

Although participants considered two of the five puberty-related recommendations to be patient-centered, opinion about assessing and treating pubertal delays varied substantially both within and between panels. Some caregivers and individuals with DMD argued that dealing with pubertal delays is not as important as handling other problems: “Puberty doesn’t matter that much compared to everything else,” said one individual with DMD. Some caregivers noted that these assessments and treatments require additional visits to specialists and may not be covered by insurance. At the same time, individuals with DMD stressed the importance of normal pubertal development and self-esteem: “I agree that heart, lungs, etc. are vital, but I would argue that our cognitive behavior and socialization is essential as well. I think fitting in is key to socialization.”

Participants agreed that assessing pubertal delays in boys with no signs of puberty by age 14 and referring them to an endocrinologist for treatment was patient-centered. However, they did not feel that assessing pubertal status using Tanner staging twice a year starting from age 9 was patient-centered. A caregiver commented: “When your child is young and in the early stages, we, caregivers, are so concerned with how long our sons will walk...or live that puberty just doesn’t play a role that young.” Other caregivers emphasized the importance of assessments: “I wish this was done for my boys, and I think the reasoning is sound. It makes a lot of sense. Seeing an endocrinologist is important, just need to make sure they are familiar with Duchenne.” Nonetheless, some participants felt that Tanner staging procedure is “very invasive for what I find of little benefit if there are no obvious signs [of pubertal delays].”

Testosterone treatment discussions highlighted the tension between addressing emotional and social well-being of individuals with DMD, the importance of ensuring normal pubertal development, and the potential of negative side effects of hormone replacement therapy, including mood swings. As an individual with DMD put it, “I feel I went through puberty a little later because of the long-term use of Prednisone. So, making sure that it isn’t causing more issues [is important]. It is a good idea to get it checked because self-esteem issues could start like with my voice being higher than most boys in my school.” A caregiver noted that “development is important because it would be embarrassing to not keep up with peers. I imagine if I were a child again knowing that I am going through the same thing my friends are would help with social development as well...” Nonetheless, participants were very concerned about side effects: “Steroids and Duchenne tend to come with the mood swings and other issues. I think adding to that may complicate behavior issues more.”

The only treatment-related recommendation that was deemed patient-centered focused on slowly increasing testosterone dosing to mimic normal pubertal development after beginning treatment. “We want to do what is best for our children and there is plenty of scientific evidence to support this recommendation,” said a caregiver.

To summarize, puberty-related recommendations are also preference-sensitive, and a child’s age may affect a family decision to follow them. Clinicians should explain the benefits and drawbacks of treating pubertal delays and engage patients and their caregivers in shared decision-making to identify the best treatment plan. Participants also noted that individuals with DMD and their caregivers have to make collaborative decisions about assessing and treating pubertal delays. The patient perspective should play an important role in decisions about pubertal development because of the unique challenges pubertal delays may pose to social development and self-esteem of individuals with DMD. According to one caregiver, “both the family and patient need to agree...patients should [not] be given something, unless they are in total agreement with the reasons, the therapy, and the potential side effects.”

## Discussion

Thematic analysis of participant comments identified several factors that affected their perceptions of patient-centeredness of the 2018 DMD care considerations. These factors are also likely to affect patient and caregiver guideline adherence. We grouped these factors into six domains that align well with the factors that also affect provider adherence to guidelines [25, 26].

### Content of recommendations

The subject and content of care guidelines can affect patient adherence. Patients and caregivers did not feel that some recommendations, including those related to vertical growth, address high priority issues As previous research shows, recommendations that do not address priority issues may have low adherence levels [27]. Participants discussed patient-centeredness of assessment recommendations more extensively than treatment recommendations (analysis not shown). Therefore, it may be beneficial for clinicians to educate patients and caregivers on the importance and benefits of assessment.

When discussing treatment recommendations, patients and caregivers tend to focus on treatment safety and efficacy. They may have doubts about recommendations that include treatment options with potentially significant side effects, uncertain efficacy, and those based on off-label use of medications. Indeed, previous research suggests that adverse drug events and patients’ fear of negative side effects can reduce their guideline adherence [28]. At the same time, we found that patients and caregivers may prefer recommendations that stress family-centeredness by explaining how patients and caregivers can work together to address a health concern.

### Format of recommendations

Patients and caregivers seem to value clear and unambiguous recommendations that offer concrete examples and do not include unreasonable expectations. Understanding what recommendation say and what activities fit well with a given recommendation can facilitate compliance. This finding is consistent with the literature on provider adherence to guidelines: providers comply with guidelines that they can easily understand and follow and do not require any specific resources [29]. Moreover, patients and caregivers appreciate recommendations that are tailored specifically to their condition and account for any functional limitations a given patient group may have. Although recommendations may be developed based on the clinical evidence collected from patients with a related condition or concern, the final wording of the recommendations should be tailored to a specific patient population. This is particularly important for rare diseases where there might be a lack of high certainty evidence [10].

### Patient characteristics

Patients’ demographic characteristics (age was of particular importance in our study) also affect compliance, which is consistent with the results of previous studies on guideline compliance [30]. In progressive conditions where patient state changes over time, patients and caregivers often focus on immediate concerns. Nonetheless, considering future needs is important to allow patients and caregivers time to prepare for future disease phases.

Other patient-level factors that may affect compliance include expected physical and psychological discomfort caused by following the recommendation (pain level, fear of needles, ability to stay still during a procedure) and logistical challenges, including time commitment and distance traveled to receive care, among others. Providers also emphasized logistical challenges as a potential patient and family barrier that had affected adherence to the earlier version of care considerations [16].

### Providers

Guideline adherence from the patient and caregiver perspective can be impacted by access to specialists and sub-specialists; provider awareness of, and agreement with, the guideline recommendation; a provider’s prior experience with a given condition; and provider qualifications and certifications. Not having access to specialists was of particular importance to our study participants. Providers with deep understanding of a given condition are more likely to be aware of the guidelines and realize their importance [29]. As a result, they are more likely to recommend guideline-adherent assessment and treatment options [31]. Agreement between the provider and patient/family regarding the importance of following a recommended guideline is also critical to adherence [32]. Engaging patients in shared decision-making helps identify care options that are evidence-based and preference-concordant; such options are more likely to be followed [33, 34].

### Social factors

A desire to lead normal lives and be accepted by peers are likely to affect adherence to those guideline recommendations that help patients fit in. Thus, positive peer pressure may have a strong influence on guideline adherence, assuming that social expectations are consistent with recommendation’s goals. The existence of a social support system can facilitate adherence by making the logistics of following recommendations easier and reducing social stigma that may be associated with certain conditions or procedures. Research on providers shows that a lack of support from peers and superiors can have a negative impact on their guideline adherence [29].

### Financial factors

From the patient and caregiver perspective, treatment costs and insurance coverage are the main factors that affected access and adherence to many recommendations. Affordability is an issue for medications that have already been approved and are in use. However, it may become a much more significant issue with exorbitant pricing of newly approved therapies in rare disease. Additionally, limiting coverage on physical and occupational therapy that payers view as “maintenance” rather than improving functioning prevents access to guideline recommended practices. The literature also supports the negative impact of high copayments and medication costs on guideline adherence [35].

In summary, factors that affect guideline compliance from the patient and caregiver perspectives are similar to those affecting provider adherence and are not limited to DMD. Providers also tend to adhere to clear guidelines that are easy to implement, grounded in strong scientific evidence, and do not require additional resources [16, 28, 29]. Although we only looked at DMD, the barriers that patients and caregivers named are also common in other conditions, including knee osteoarthritis [27], diabetes [28], and cardiovascular disease [31], among others. Consistency between the outcomes a recommendation is designed to address and patient preferences is key [32], regardless of whether patients are children or adults or have a common or a rare condition.

### Limitations

We note several limitations of our study. First, although our sample included both patients and caregivers, it was purposive. Therefore, our conclusions may not represent the opinions and experiences of Duchenne families in general. However, purposive sampling is common for expert panels and the Delphi method [36] because the goal is to include the most knowledgeable individuals. Second, our measure of patient-centeredness was developed specifically for this project. Although it is consistent with the GRADE Evidence to Decision Framework [37] and was pilot-tested as part of our project, it should be formally validated. Finally, we identified the factors that may affect patient and caregiver adherence to guidelines based on one condition only. Although we believe that these factors are likely to affect guideline adherence across the conditions, it is possible that additional factors affecting guideline compliance may emerge from the experiences of patients with other conditions, including chronic diseases. Therefore, our engagement approach should be validated further in other clinical conditions.

## Conclusions

This study demonstrates a new way to include the patient and caregiver voice in developing care guidelines and to identify the factors that can affect their guideline adherence. Using this methodology across diagnoses could increase the patient voice in guideline development, facilitate shared decision-making, and ultimately enhance patient and family adherence to care recommendations. The results of this work contribute to a growing literature on the factors that patients and caregivers think affect their guideline adherence. Understanding consistency between provider- and patient/caregiver-identified barriers to following guideline recommendations can lead to developing more successful interventions for increasing guideline adherence.

## Additional file

Additional file 1: Appendix: Endocrine and Bone Health Care Recommendations Included in Our Study. (PDF 191 kb)

## Data Availability

The data analyzed during this study are available from the corresponding authors on reasonable request.

## Abbreviations

DMD: Duchenne Muscular Dystrophy
PPMD: Patient project muscular dystrophy
RAM: RAND/UCLA Appropriateness method
