# Supplementary material for: Patient and caregiver perspectives on guideline adherence: the case of endocrine and bone health recommendations for Duchenne muscular dystrophy
Source: Orphanet J Rare Dis. 2019 Aug 20;14:205. doi: 10.1186/s13023-019-1173-7 (PMC6702715; doi:10.1186/s13023-019-1173-7)
Supplement: Supplementary file 1 — Appendix: Endocrine and Bone Health Care Recommendations Included in Our Study. (PDF 191 kb) [file 13023_2019_1173_MOESM1_ESM.pdf]

Appendix: Endocrine and Bone Health Care Recommendations Included in Our Study

Note: Recommendations presented in red font did not meet our patient-centeredness criteria.

Vertical Growth

Recommendation 1: Assessment of growth

Height and length measurements for patients with Duchenne should be assessed every 6 months until puberty is complete and final height is reached.

Clinical reason for recommendation: To identify any growth delays early on by comparing individual's height to the height of children of similar age.

Process: Track height/length on a standard growth chart twice a year until puberty.

Additional information: Height and length measurements are typically taken during a routine health visit, and should be tracked every 6 months until puberty/final adult height is reached.

Recommendation 2a: Identification of impaired growth (patients 7 years of age and younger)

An x-ray of the left hand and wrist to assess bone age should be performed in patients 7 years of age and younger with suspected impaired growth.

Clinical reason for recommendation: To identify signs of impaired growth early on by comparing individual's bone growth to children of the same age.

Process: Take an X-ray of the left hand and wrist to measure bone growth/skeletal maturity when you or your doctor suspect impaired growth.

Additional information: X-rays of the bones are used to assess bone growth and the closure of growth plates in the hand in order to determine how mature/immature the skeleton is and how much longer growth may be possible for a child of a given age. X-rays create pictures of your bones in a painless manner.

Recommendation 2b: Identification of impaired growth (patients between 8 and 12 years of age)

An x-ray of the left hand and wrist to assess bone age should be performed in patients between 8 and 12 years of age with suspected impaired growth.

Clinical reason for recommendation: To identify signs of impaired growth early on by comparing individual's bone growth to children of the same age.

Process: Take an X-ray of the left hand and wrist to measure bone growth/skeletal maturity when you or your doctor suspect impaired growth.

Additional information: X-rays of the bones are used to assess bone growth and the closure of growth plates in the hand, in order to determine how mature/immature the skeleton is and how much longer growth may be possible for a child of a given age. X-rays create pictures of your bones in a painless manner.

Recommendation 2c: Identification of impaired growth (patients between 13 and 18 years of age)

An x-ray of the left hand and wrist to assess bone age should be performed in patients between 13 and 18 years of age with suspected impaired growth.

Clinical reason for recommendation: To identify signs of impaired growth early on by comparing individual's bone growth to children of the same age.

Process: Take an X-ray of the left hand and wrist to measure bone growth/skeletal maturity when you or your doctor suspect impaired growth.

Additional information: X-rays of the bones are used to assess bone growth and the closure of growth plates in the hand, in order to determine how mature/immature the skeleton is and how much longer growth may be possible for a child of a given age. X-rays create pictures of your bones in a painless manner.

Recommendation 3: Identification of reasons for impaired growth and development of a treatment plan

Patients should be referred to an endocrinologist for assessment if they are not progressing on their growth curves, have gained less than approximately 1.5 inches of height per year, or are in the 3rd percentile of height for their age.

Clinical reason for recommendation: To determine the reasons for impaired growth and to develop an individualized treatment plan.

Process: A consultation with an endocrinologist (hormone doctor) for all patients who are not growing as expected (gained less than approximately 1.5 inches a year or are in the 3rd percentile of height for their age).

Additional information: Children/adolescents should grow at least approximately 1.5 inches a year and should be as tall as, or taller than, 3% of other children in this age group. If growth is

not occurring at this rate, a consultation with an endocrinologist is needed to determine the reason for the impaired growth.

Recommendation 4a: Treatment of impaired growth with growth hormone therapy (patients 7 years of age and younger)

Recombinant human growth hormone (rhGH) should be reserved for treating children 7 years of age or younger only if they had abnormal growth hormone stimulation tests due to the inconclusive evidence about its effectiveness.

Clinical reason for recommendation: Recombinant growth hormone (rhGH), a laboratory produced growth hormone, should only be used for treating children with Duchenne who have short stature and a confirmed persistent shortage of naturally produced growth hormone. It should not be used routinely to treat Duchenne-related growth failure.

Process: Growth hormone therapy may require daily injections.

Additional information: While rhGH is recommended for the treatment of growth delay related to growth hormone deficiency, it is currently not recommended for growth delays in Duchenne unrelated to growth hormone deficiency. There is no conclusive evidence that shows that routine use of rhGH is effective in treating Duchenne-related growth delays.

Recommendation 4b: Treatment of impaired growth with growth hormone therapy (patients between 8 and 12 years of age)

Recombinant human growth hormone (rhGH) should be reserved for treating children between 8 and 12 years of age only if they had abnormal growth hormone stimulation tests due to the inconclusive evidence about its effectiveness.

Clinical reason for recommendation: Recombinant growth hormone (rhGH), a laboratory produced growth hormone, should only be used for treating children with Duchenne who have short stature and a confirmed persistent shortage of naturally produced growth hormone. It should not be used routinely to treat Duchenne-related growth failure.

Process: Growth hormone therapy may require daily injections.

Additional information: While rhGH is recommended for the treatment of growth delay related to growth hormone deficiency, it is currently not recommended for growth delays in Duchenne unrelated to growth hormone deficiency. There is no conclusive evidence that shows that routine use of rhGH is effective in treating Duchenne-related growth delays.

Recommendation 4c: Treatment of impaired growth with growth hormone therapy (patients between 13 and 18 years of age)

Recombinant human growth hormone (rhGH) should be reserved for treating children between 13 and 18 years of age only if they had abnormal growth hormone stimulation tests due to the inconclusive evidence about its effectiveness.

Clinical reason for recommendation: Recombinant growth hormone (rhGH), a laboratory produced growth hormone, should only be used for treating children with Duchenne who have short stature and a confirmed persistent shortage of naturally produced growth hormone. It should not be used routinely to treat Duchenne-related growth failure.

Process: Growth hormone therapy may require daily injections.

Additional information: While rhGH is recommended for the treatment of growth delay related to growth hormone deficiency, it is currently not recommended for growth delays in Duchenne unrelated to growth hormone deficiency. There is no conclusive evidence that shows that routine use of rhGH is effective in treating Duchenne-related growth delays.

Weight Management

Recommendation 1: Maintaining healthy weight (diet)

At the time of glucocorticoid initiation, the nutritionist should address an individual's diet by emphasizing family centered health eating, including:

Control portions of meats and starches,
Increase fruit/vegetables,
Decrease sugar-containing drinks and increase water intake,
Choose low-fat or fat-free milk,
Limit fast food and high-calorie/salty snacks (chips, cookies, etc.),
Choose low-sodium versions of processed foods, limit added salt in cooking and at the table, and use available salt substitutes,
Choose whole-grain/low-sugar cereals, breads, etc.

Clinical reason for recommendation: To prevent/manage weight gain during glucocorticoid use.

Process: Education of Duchenne families regarding healthy eating habits, maintaining healthy nutrition, and preventing obesity delivered by nutritionists before initiating glucocorticoid therapy.

Additional information: Glucocorticoid therapy includes administration of steroid hormones (prednisone or emflaza). These medications can increase appetite, which may result in unhealthy eating and weight gain.

Recommendation 2: Maintaining healthy weight (nutrition)

At the time of glucocorticoid initiation, the nutritionist should create a general nutritional plan based on the total energy expenditure (TEE) that includes specific recommendations for calorie, protein and fluid intake.

Clinical reason for recommendation: To prevent/manage weight gain during glucocorticoid use.

Process: Development of an individualized nutritional plan by nutritionists based on the number of calories an individual with Duchenne burns daily before initiating glucocorticoid therapy.

Additional information: Having an individualized nutritional plan may help individuals with Duchenne and their families better understand the importance of developing healthy eating habits, maintaining healthy nutrition, and preventing obesity, dehydration and constipation, which are common side effects of glucocorticoid therapy.

Recommendation 3: Maintaining healthy weight (exercise)

At the time of glucocorticoid initiation, the physical therapist should emphasize family-centered physical activity (adapted in terms of amount/duration/frequency as necessary and as recommended by care team to meet the needs of the individual with Duchenne as his ambulation declines).

Clinical reason for recommendation: To prevent/manage weight gain during glucocorticoid use.

Process: Education of Duchenne families regarding appropriate exercise activities delivered by physical therapists before initiating glucocorticoid therapy.

Additional information: Education on appropriate physical activity may help patients maintain healthy lifestyle and prevent obesity while taking glucocorticoid therapy. Due to declining physical ability over time, individuals with Duchenne will find it more difficult to exercise to maintain appropriate weight.

Bone Health

Recommendation 1: Assessment of spinal health and detection of vertebral compression fractures

Baseline spinal imaging should be performed in all boys with Duchenne using lateral spinal x-rays.

Clinical reason for recommendation: To detect vertebral fractures early on and to assess bone health.

Process: Assessment of the height and compression of vertebral bodies (bones of the spine) using lateral spinal x-rays. Spinal x-rays are pictures of the spine that help find injuries or diseases that affect the discs or joints of the spine. X-rays are relatively quick to perform but require patients to lie on an x-ray table.

Additional information: Vertebral bodies may not be compressed, or may be mildly, moderately, or severely compressed. Mild compression may be a sign that the patient is at risk to develop vertebral fractures; moderate or severe compression may be a sign of vertebral fractures that are present now. Vertebral fractures may occur with or without back pain, so all boys with Duchenne should have their spine imaged to create baseline information about spinal health even if they are not having pain.

<u>Recommendation 2: Assessment of bone health/mineralization using dual energy X-ray absorptiometry (DEXA) scans</u>

Individuals with known risk factors for osteoporosis, including loss of ambulation and/or glucocorticoid therapy, should undergo intermittent long bone imaging to evaluate changes in the density of the long bones.

Clinical reason for recommendation: To assess the density of the long bones by comparing to the known standard for people who do not have bone health concerns and are of a similar age.

Process: Regular assessment (once every 1-3 years) of the mineralization of long bones in individuals on glucocorticoid therapy and non-ambulatory individuals using dual energy X-ray absorptiometry (DEXA) scans.

Additional information: Healthy bones are dense, mineralized, and strong. Glucocorticoids may reduce the amount of minerals deposited in the bones, resulting in weak or thinning bones (osteoporosis). DEXA scans are used to assess the density and mineralization of bones and determine the strength of bones, as well as the likelihood of fracture. Like most x-rays, DEXA scans are relatively quick to perform and are safe and painless.

<u>Recommendation 3: Treatment of vertebral fractures and bone loss with IV bisphosphonates</u>

An intravenous (IV) bisphosphonate should be considered in individuals with Duchenne who have had low-trauma long bone fractures or vertebral fractures, or who have been shown to have moderate or severe vertebral compression on lateral spinal x-ray, with or without back pain, indicating the presence of vertebral fractures.

Clinical reason for recommendation: To treat vertebral fractures and back pain to prevent future long bone and vertebral fractures.

Process: Administration of IV bisphosphonates (a class of drugs that prevent the loss of bone mass) to patients with low-trauma long bone fractures (i.e., fractures that occurs without a fall), vertebral fractures, or moderately or severely vertebral compression, with or without complaints of back pain.

Additional information: Because bisphosphonates remain off label for children in most countries, anyone experiencing a low-trauma fracture of a long bone before the age of 18 should consult with an expert in osteoporosis management who can help ensure proper bisphosphonate type, dosing, duration of treatment and monitoring of treatment efficacy and safety during treatment.

Puberty

Recommendation 1: Assessment of puberty

Pubertal status should be assessed by Tanner staging every 6 months, starting by 9 years of age.

Clinical reason for recommendation: To detect early delays in puberty.

Process: Assessment of biological maturation of individuals with Duchenne twice a year, starting by age 9, using the Tanner Scale.

Additional information: The Tanner Scale defines stages of puberty using such characteristics as the size of genitals, testicular volume, and development of pubic hair. This assessment of pubertal status may require a genital examination by a healthcare provider, which may be embarrassing to boys.

Recommendation 2: Assessment and treatment of delays in puberty

Patients with no signs of puberty by age 14 (i.e., testicular volume <4 cubic cm by age 14) should be referred to endocrinology for assessment.

Clinical reason for recommendation: To assess and treat pubertal delays.

Process: Individuals with Duchenne without any signs of puberty (i.e., testicular volume) are referred to an endocrinologist for further assessment and to discuss treatment options.

Additional information: Puberty should start by age 14 years. If by 14 years there have been no signs of puberty starting, a visit to an endocrinologist is necessary to determine the cause of delayed puberty.

Recommendation 3: Use of testosterone replacement therapy to treat delays in puberty

Testosterone replacement therapy should be used for treating individuals with Duchenne older than 14 years of age who have no signs of starting puberty and/or who have confirmed hypogonadism (low levels of testosterone).

Clinical reason for recommendation: To initiate pubertal development.

Process: Initiation of testosterone replacement therapy in individuals with Duchenne who (1) are older than 14, (2) do not have any signs of puberty, and/or (3) who have low levels of testosterone, a hormone that controls male puberty. Patients with a delayed onset of puberty should be given testosterone, either by injection or topical gel to aid the start of start of pubertal development.

Additional information: Although testosterone therapy can help initiate pubertal development, it may also bring on typical male teenage behaviors, such as mood swings and more interest in sex, as well as physical changes, such as the development of acne and body odor.

Recommendation 4: Use of testosterone therapy while taking glucocorticoids

Testosterone replacement therapy can be considered in boys older than 12 years taking glucocorticoids with absent pubertal development.

Clinical reason for recommendation: To initiate pubertal development.

Process: Clinicians should consider prescribing testosterone to individuals with Duchenne who are (1) older than 12, (2) do not have any signs of puberty, and (3) take glucocorticoids, which are man-made versions of steroid hormones used to slow the progression of muscle weakness.

Additional information: Testosterone therapy may bring on typical male teenage behaviors, such as mood swings and more interest in sex, as well as physical changes, such as the development of acne and body odor.

Recommendation 5: Testosterone dosing to mimic normal pubertal development

Testosterone therapy should be initiated at a low dose and slowly increased to adult replacement doses over several years in an attempt to mimic normal pubertal development.

Clinical reason for recommendation: Mimicking of normal pubertal development.

Process: Clinicians should start testosterone therapy in boys with Duchenne slowly with a low pediatric dose, which should then be increased to an adult level over the course of several years.

Additional information: Testosterone therapy may bring on typical male teenage behaviors, such as mood swings and more interest in sex, as well as physical changes, such as the development of acne and body odor.
